# Perspectives on joint EANM/SNMMI/ANZSNM practice guidelines/procedure standards for [^18^F]FDG PET/CT imaging during immunomodulatory treatments in patients with solid tumors

**DOI:** 10.1186/s40644-022-00512-z

**Published:** 2022-12-20

**Authors:** E. Lopci, N. Aide, A. Dimitrakopoulou-Strauss, L. Dercle, A. Iravani, R. D. Seban, C. Sachpekidis, O. Humbert, O. Gheysens, A. W. J. M. Glaudemans, W. A. Weber, A. D. Van den Abbeele, R. L. Wahl, A. M. Scott, N. Pandit-Taskar, R. J. Hicks

**Affiliations:** 1 Nuclear Medicine Unit, IRCCS – Humanitas Research Hospital, Via Manzoni 56, 20089 Rozzano, MI Italy; 2 Nuclear Medicine Department, University Hospital, Caen, France; 3 INSERM ANTICIPE, Normandie University, Caen, France; 4 Clinical Cooperation Unit Nuclear Medicine, German Cancer Research Center (DKFZ), Im Neuenheimer Feld 280, 69210 Heidelberg, Germany; 5 Department of Radiology, New York Presbyterian, Columbia University Irving Medical Center, New York, NY USA; 6 Department of Radiology, The University of Washington, Seattle, USA; 7 Fred Hutchinson Cancer Center, Seattle, USA; 8 Department of Nuclear Medicine and Endocrine Oncology, Institut Curie, 92210 Saint-Cloud, France; 9 Laboratoire d’Imagerie Translationnelle en Oncologie, Inserm, Institut Curie, 91401 Orsay, France; 10 Department of Nuclear Medicine, Centre Antoine-Lacassagne, Université Côte d’Azur, Nice, France; 11 TIRO-UMR E 4320, Université Côte d’Azur, Nice, France; 12 Department of Nuclear Medicine, Cliniques Universitaires Saint-Luc, Université Catholique de Louvain (UCLouvain), Brussels, Belgium; 13 Nuclear Medical Imaging Center, Department of Nuclear Medicine and Molecular Imaging, University of Groningen, University Medical Center Groningen, Groningen, The Netherlands; 14 Department of Nuclear Medicine, Klinikum rechts der Isar, Technical University Munich, Ismaninger Str. 22, 81675 Munich, Germany; 15 Department of Imaging, Dana-Farber Cancer Institute and Department of Radiology, Mass General Brigham Hospitals, Harvard Medical School, Boston, MA USA; 16 Mallinckrodt Institute of Radiology, Washington University School of Medicine, St. Louis, MO USA; 17 Department of Molecular Imaging and Therapy, Austin Health, Studley Rd, Heidelberg, VIC 3084 Australia; 18 Olivia Newton-John Cancer Research Institute, Heidelberg, Australia; 19 Faculty of Medicine, University of Melbourne, Melbourne, Australia; 20 School of Cancer Medicine, La Trobe University, Melbourne, Australia; 21 Molecular Imaging and Therapy Service, Department of Radiology, Memorial Sloan-Kettering Cancer Center, 1275 York Ave, New York, NY 10065 USA; 22 Weill Cornell Medical College, New York, NY 10065 USA; 23 The Department of Medicine, St Vincent’s Medical School, the University of Melbourne, Melbourne, Australia

**Keywords:** Positron emission tomography, PET/CT, [^18^F]FDG, Guideline, Immunotherapy, Treatment response, Malignant tumors, precision medicine

## Abstract

Response assessment in the context of immunomodulatory treatments represents a major challenge for the medical imaging community and requires a multidisciplinary approach with involvement of oncologists, radiologists, and nuclear medicine specialists. There is evolving evidence that [^18^F]FDG PET/CT is a useful diagnostic modality for this purpose. The clinical indications for, and the principal aspects of its standardization in this context have been detailed in the recently published “*Joint EANM/SNMMI/ANZSNM practice guidelines/procedure standards on recommended use of [*^*18*^*F]FDG PET/CT imaging during immunomodulatory treatments in patients with solid tumors version 1.0*”. These recommendations arose from a fruitful collaboration between international nuclear medicine societies and experts in cancer treatment. In this perspective, the key elements of the initiative are reported, summarizing the core aspects of the guidelines for radiologists and nuclear medicine physicians. Beyond the previous guidelines, this perspective adds further commentary on how this technology can advance development of novel therapeutic approaches and guide management of individual patients.

The recently published “*Joint EANM/SNMMI/ANZSNM practice guidelines/procedure standards on recommended use of [*^*18*^*F]FDG PET/CT imaging during immunomodulatory treatments in patients with solid tumors version 1.0*” [1] provide guidance for nuclear medicine specialists on how to correctly perform, interpret and report [^18^F]FDG PET/CT in patients with solid tumors undergoing treatment with immune checkpoint inhibitors. While the original manuscript is freely available, we feel that the wider cancer imaging community, who aren’t necessarily themselves nuclear medicine specialists, should be aware of the key elements of these guidelines and of the importance of multidisciplinary involvement in the appropriate management of patients undergoing this increasingly common treatment modality.

In the context of recognized limitations of standard radiological response assessment, several studies over the last decade, despite being primarily focused on melanoma and non-small cell lung cancer [2], have provided evidence supporting the use of [^18^F]FDG PET/CT imaging for monitoring immunomodulatory treatments. However, a lack of multicentre randomized trials designed to prove the clinical impact of the modality has resulted in heterogeneous application across cancer types. Initial recommendations for assessing tumor response and reporting immune-related adverse effects (irAEs) were published following a symposium held during the 2017 Annual Congress of the European Association of Nuclear Medicine [3]. Subsequently, several adaptations of metabolic response criteria have been proposed [2–7], but there has been a lack of consensus on the appropriate use of this diagnostic modality. Consequently, a joint international initiative involving major nuclear medicine societies was coordinated to provide recommendations for use by professionals directly and indirectly involved in [^18^F]FDG PET/CT imaging in the course of immunomodulatory treatment. This resulted in the abovementioned joint practice guidelines and procedural standards [1]. Importantly, the guidelines detail the key aspects of [^18^F]FDG PET/CT interpretation at various phases of the treatment pathway (Fig. 1). Further, we believe that there is an opportunity to embed molecular imaging in the development of novel immune therapies, which is currently an extremely active research domain.

**Fig. 1:**
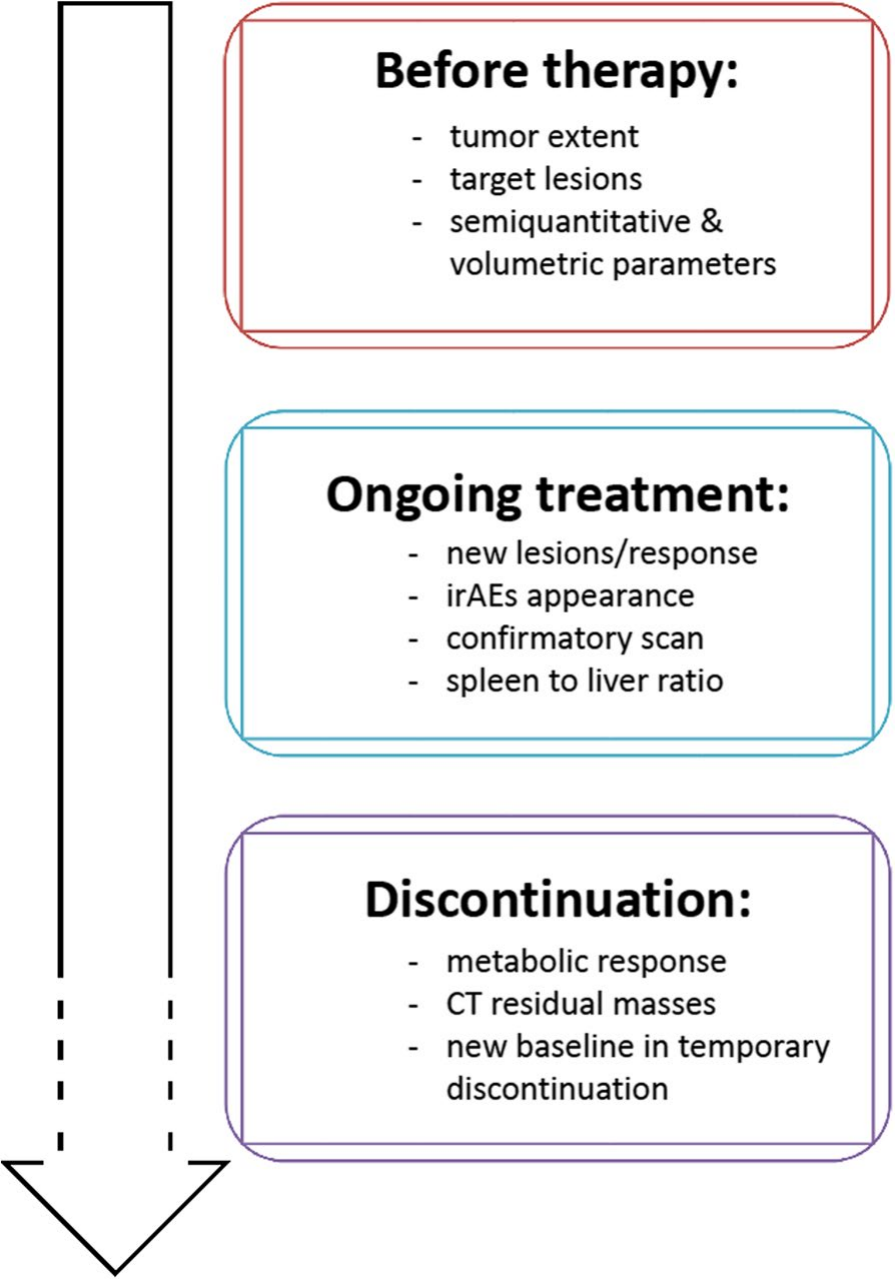
Timeline of [^18^F]FDG PET/CT interpretation during immunomodulatory treatment of solid tumors

## Summary of key recommendations

[^18^F]FDG PET/CT is recommended as a baseline before treatment is started since it provides tumor stage and defines extent prior to the treatment. This also allows the target lesions to be assigned for subsequent response assessment. Additionally, advanced imaging biomarkers can be derived from baseline scan. These include semiquantitative and volumetric parameters, which can be useful to guide clinical decisions in patients who subsequently demonstrate atypical response patterns. Baseline metabolic tumor volume (MTV) is increasingly recognized to be an important prognostic and possibly predictive biomarker of response.

Interim response evaluation with [^18^F]FDG PET/CT is recommended routinely after 3–4 cycles of immunotherapy, or earlier in the case of discordant findings obtained from CT imaging or suspicion of disease progression due to clinical deterioration. Available metabolic response criteria can be used [1–9], recognizing that differentiation between disease progression and pseudoprogression requires a follow-up scan 4–8 weeks later in the setting of clinical stability, emphasizing the importance of open communication with the managing clinician. Alternatively, a biopsy of the radiographically/metabolically progressive lesion may be indicated. Herein, a description of the signs of immune activation, such as increased spleen to liver ratio or increased activity in nodes in the drainage basin of previously documented metastatic sites, is considered helpful. Not forgetting that the occurrence of irAEs documented on [^18^F]FDG PET/CT at any time point must be described and reported, while severe cases should be promptly communicated to referring clinicians as several complications can be life-threatening. These particularly include colitis and pneumonitis.

At, or before treatment discontinuation of immune checkpoint therapy, a [^18^F]FDG PET/CT may be also obtained to confirm metabolic response, especially in patients with a partial response or stable disease on CT. The prognostic impact of a complete metabolic response at this time point is emphasized in several studies [1]. While in patients requiring a temporary interruption of immunotherapy, a new baseline [^18^F]FDG PET/CT for restaging is recommended before restarting treatment.

Since general recommendations for acquisition procedures and protocols, documentation and reporting for [^18^F]FDG PET/CT have already been detailed in the EANM/SNMMI practice guidelines/procedure standards for tumor imaging [10, 11], this joint initiative focused on the special considerations to be kept in mind during immunomodulatory treatments and are summarized in Table 1.

**Table 1:** Key points to consider during [^18^F]FDG PET/CT procedure and reporting

	Special alerts	Standard reference
Protocol/procedure	The skull base should be included in the imaging field-of-view to evaluate possible immune-related hypophysitis. Whole-body imaging from the vertex to the feet is recommended in neoplasia with tendency to extensive metastatic disease (e.g., melanoma, Merkel cell tumor, etc.).	EANM guideline [10] and SNMMI procedure standards for tumor imaging [11]. The RSNA QIBA FDG/CT guidance [12] and specific radiologic society guidelines for contrast-enhancement [^18^F]FDG PET/CT [13] International harmonizing standards, i.e. EANM/EARL program [10, 14].
Reporting/documentation	Type and number of cycles of immunotherapy must be specified. Target lesions and response pattern to be reported based on the chosen metabolic response criteria, which should be recorded [4–9]. Quantitation of metabolic tumor burden is recommended. Comparison with relevant morphologic findings on CT, and request for confirmatory scanning in case of suspected progression. Appearance, extent, severity, and variation over time of the irAEs and other signs of immune activation must be reported.	

Abbreviations: *RSNA* Radiological Society of North America, *QIBA* Quantitative Imaging Biomarkers Alliance, *EARL* EANM Research Ltd., *irAEs* immune-related adverse events

## Future perspectives

Immunotherapy assessment with [^18^F]FDG PET/CT represents a dynamic field of research. Therefore, the abovementioned practice guidelines/procedure standards should not be considered as fixed, but rather as a current guidance on how to perform [^18^F]FDG PET/CT studies in patients undergoing immunomodulatory treatments that might be modified by new evidence.

One of the aspects that will necessarily become increasingly relevant in the future for patient selection and response prediction is represented by the novel immune-PET tracers that could be combined with [^18^F]FDG PET/CT for molecular imaging phenotyping, particularly for selecting therapeutic agents, or combinations thereof, and providing differentiation of pseudoprogression from true progression. Several of these tracers, such as the radiolabeled immunotherapeutic antibodies, anti-CD8, AraG, granzyme B, and others may add specificity to the modality resulting in: 1) better characterization of the entire tumor and its degree of heterogeneity in one setting; 2) prognostic markers that may characterize the tumor and its microenvironment as immune-rich from poor, which may help guide the therapeutic choice; 3) predictive markers after initiation of therapy to differentiate immune response from progression, while avoiding the need for repeat imaging; and, 4) early detection of immune adverse events early before the patient becomes symptomatic, or has biochemical evidence of toxicity as to initiate timely therapy. The toolbox of novel radiopharmaceuticals for evaluation of the immune microenvironment has been recently reviewed [15], and possible algorithms for incorporating these into treatment planning in combination with [^18^F]FDG PET/CT have been proposed [16] .

While awaiting clinical validation of the abovementioned immune-PET tracers, an adequate awareness on how to utilize [^18^F]FDG PET/CT should be part of the basic knowledge-base of oncologists involved in delivering immunotherapy and is vital for cancer imaging specialists. As with many other clinical indications in nuclear medicine, a multidisciplinary approach is important to provide clinical context when imaging findings raise the possibility of pseudoprogression or hyperprogression or irAEs are suspected. In the latter case, open communication channels with the managing clinician are critical to optimally manage unexpected events. In view of the complexity of new therapies and often unique imaging patterns on [^18^F]FDG PET, which have recently been reviewed in this journal from the perspective of malignant melanoma management [17], it is vital that prospective clinical research and trials are conducted to establish evidence to appropriately guide nuclear medicine specialists and clinicians in managing their patients. Premature cessation of effective therapy or continuation in the face of life-threatening complications can have serious consequences for patients, including premature death as well as both acute and chronic sequelae [18], and therefore abundant caution and effective communication between clinicians is needed.

## Incorporating metabolic imaging into trials of novel immunotherapy regimens

There are increasing therapeutic options that modify the immune microenvironment that are competing for clinical application alone or in combination with existing approved agents. These range from immune priming agents through to immune checkpoint inhibitors, with these also being combined with targeted agents, chemotherapy and radiotherapy creating significant issues with respect to regulatory approvals and comparison of trial outcomes [19]. Further, combination therapies may introduce unique patterns of response and new toxicities, providing challenges for designing optimal treatment regimens [20]. For example, radiation exposure may increase neoantigenic presentation and has potential implications for the combination of radionuclide therapy with immune checkpoint inhibitors as an evolution of emerging theranostic paradigms with respect to administered activity and dosing intervals [21], but may also sensitize normal organs exposed to radiation to irAEs.

Given increasing evidence of the importance of metabolic tumor volume [22], randomized clinical trials comparing treatment regimens should ideally be stratified and incorporation of [^18^F]FDG PET/CT into therapeutic response assessment may yield earlier readouts of superior efficacy with a complete metabolic response having favorable prognostic implications even in patients with stable or partial radiologic responses.

## Conclusions

The recently published guidelines for the use of [^18^F]FDG PET/CT in the context of immune modulatory therapies provide a starting point for more routine implementation of this technology in improving selection and monitoring of patients receiving these therapies. Cancer imaging specialists should be aware of the recommendations and enter an active dialogue with their clinical colleagues delivering these treatments both in a general educational sense and in respect to the results of findings in individual patients. Significant opportunity exists for incorporating [^18^F]FDG PET/CT into clinical trial designs to assess evolving treatment combinations in this dynamic field of clinical research.

## Data Availability

Not applicable.
